# Preliminary results from minimally invasive video-assisted thyroidectomy

**DOI:** 10.1590/S1516-31802005000600011

**Published:** 2005-11-01

**Authors:** Rogério Aparecido Dedivitis, André Vicente Guimarães

**Keywords:** Thyroidectomy, Endoscopy, Thyroid gland, Surgery, Tireoidectomia, Endoscopia, Glândula tireóide, Cirurgia

## Abstract

**CONTEXT AND OBJECTIVE:**

Minimally invasive video-assisted gasless thyroidectomy (MIVAT) has mainly been described in Italy and has been demonstrated to be a safe procedure with additional advantages regarding cosmetic results and postoperative outcome. The aim of this work is to analyze our preliminary results from minimally invasive video-assisted thyroidectomy.

**DESIGN AND SETTING:**

Retrospective study at the Head and Neck Surgery Service of Hospital Ana Costa, Santos.

**METHODS:**

Twelve patients underwent hemithyroidectomy and another three underwent total thyroidectomy by means of minimally invasive video-assisted thyroidectomy between June and September 2004. Gender, age, goiter volume, major diameter of the dominant nodule, duration of surgery, pain complaints during the first postoperative day, length of hospital stay, cosmetic result and complications were retrospectively analyzed.

**RESULTS:**

All the patients were women, with median age of 34. The median goiter volume was 16.5 ml, and the median major diameter of the nodule was 2.3 cm. Ten patients reported mild pain at the surgical site. The median scar size was 2.0 cm and all patients considered the cosmetic results excellent. The median duration of surgery was 55 minutes, all patients were discharged on the first postoperative day, and there were no complications.

**CONCLUSIONS:**

The outcome from minimally invasive video-assisted thyroidectomy is good in terms of cosmetic results, analgesia and postoperative recovery. The scar is shorter than in the conventional procedure.

## RESUMO

**CONTEXTO E OBJETIVO:**

A tireoidectomia minimamente invasiva vídeo-assistida sem insuflação de gás foi descrita principalmente na Itália e demonstrou-se um procedimento seguro com vantagens adicionais quanto aos resultados estéticos e evolução pós-operatória. O objetivo deste trabalho é analisar os nossos resultados preliminares da realização, da tireoidectomia minimamente invasiva vídeo-assistida.

**TIPO DE ESTUDO E LOCAL:**

Estudo retrospectivo realizado no Serviço de Cirurgia de Cabeça e Pescoço do Hospital Ana Costa, Santos.

**MÉTODOS:**

12 pacientes foram submetidos a hemitireoidectomia e três a tireoidectomia total, através da tireoidectomia minimamente invasiva vídeo-assistida, no Serviço de Cirurgia de Cabeça e Pescoço do Hospital Ana Costa, Santos, de junho a setembro de 2004. Gênero, idade, volume do bócio, maior diâmetro do nódulo dominante, tempo cirúrgico, queixa de dor durante o primeiro dia de pós-operatório, tempo para alta hospitalar, resultados estéticos e complicações foram retrospectivamente analisados.

**RESULTADOS:**

Todos os pacientes eram mulheres, com mediana etária de 34 anos. A mediana do volume do bócio foi de 16,5 ml, enquanto a mediana do maior eixo do nódulo dominante foi de 2,3 cm. Dez pacientes relataram dor discreta no sítio cirúrgico. A mediana da cicatriz era de 2,0 cm e todas consideraram os resultados estéticos excelentes. A mediana de tempo cirúrgico foi de 55 minutos. Todas receberam alta no primeiro dia de pós-operatório. Não houve complicações.

**CONCLUSÕES:**

A tireoidectomia minimamente invasiva vídeo-assistida proporciona bons resultados estéticos, analgesia e recuperação durante o período pós-operatório. O tamanho da cicatriz é menor que o da tireoidectomia convencional.

## INTRODUCTION

Minimally invasive video-assisted gasless thyroidectomy (MIVAT) has mainly been described in Italy^1,2^ and has been demonstrated to be a safe procedure with additional advantages regarding cosmetic results and postoperative outcome. Its results are comparable to those from the conventional procedure, while some advantages have already been demonstrated, with regard to the following aspects:^3^ shorter scar length; better analgesia and reduced edema; and better postoperative recovery. The duration of surgery is shorter than for other endoscopic techniques and comparable to the duration of conventional thyroidectomy. The mean duration of surgery has been described as 30.4 minutes for the lobectomy (ranging from 20 to 140 minutes) and 50.2 minutes for the total thyroidectomy (ranging from 35 to 140 minutes), while the mean hospital stay was 1.28 days (ranging from 1 to 4 days).^4^ The objective of the present study was to analyze our preliminary results from minimally invasive video-assisted thyroidectomy.

## METHODS

### Type of study

This was a retrospective chart review and it was approved by the research ethics committee of Universidade Metropolitana de Santos, under protocol no. 031/2004.

### Setting

Head and Neck Surgery Service, Hospital Ana Costa, Santos, State of São Paulo, Brazil.

### Sample

Twelve consecutive patients underwent hemithyroidectomy due to nodular disease and another three underwent total thyroidectomy, including two as treatment for papillary carcinoma, by means of minimally invasive video-assisted thyroidectomy from June to September 2004. The inclusion criteria for the patients were based on the following aspects:^5^ 35 mm as the maximum diameter of the nodule; 20 ml as the maximum total thyroid volume; and absence of echographic and laboratory test signs of thyroiditis.

### Procedures

Our procedure was based on the technique described by Miccoli et al.,^6^ with modifications. A zero-degree, 5 mm Storz^®^ endoscope was used. Hemostasis of the upper pedicle of the thyroid was achieved using the UltraCision ultrasonic scalpel and Ethicon^®^ CS14C device. No drainage was left in any case. Cyanoacrylate sealant was used for skin closure.

### Main measurements

Gender, age, goiter volume, major diameter of the dominant nodule, duration of surgery, pain complaints during the first postoperative day, length of hospital stay, cosmetic result and complications (vocal fold palsy, infection, hematoma and clinical signs of hypoparathyroidism) were analyzed. Postoperative pain was assessed within the first 24 hours by means of a semi-quantitative self-evaluation from 0 to +++. All the patients received endovenous dipyrone every six hours. The vocal fold mobility was evaluated by means of videolaryngoscopy performed in the office between the seventh and tenth postoperative day. The cosmetic result was evaluated by the patients themselves according the following criteria: acceptable, good and excellent.

## RESULTS

All the patients were women, with ages ranging from 25 to 68 years (median of 34). The median goiter volume was 16.5 ml (range: 11.1 to 20 ml), while the median major diameter of the nodule was 2.3 cm (range: 0.6 to 3.0 cm). The median duration of surgery was 55 minutes (range: 45 to 125 minutes). All the patients were discharged on the first postoperative day. Ten patients reported mild pain at the surgical site and five said they did not have any pain, according to the semiquantitative self-evaluation. The median scar length was 2.0 cm (range: 2 to 2.5 cm). All the patients considered the cosmetic results excellent and there were no complications.

## DISCUSSION

This procedure requires a skilled surgical team and is therefore demanding in nature. Moreover, specific instruments and optical devices are needed, which represent a high cost. However, since the technique is gasless, laparoscopic instrumentation that would result in additional charge is not necessary. The abovementioned patient inclusion criteria establish boundaries for the indication of this procedure.

The harmonic scalpel made it possible for the procedures to be performed quickly, because this device has the ability to safely and expeditiously control the feeding vessels through a limited field. As we still are on a learning curve regarding the minimally invasive video-assisted thyroidectomy procedure, it can be supposed that our surgical team will improve its performance with increasing experience. None of the procedures had to be converted into the conventional procedure. Conversion to traditional cervicotomy was required in 1.4% of the cases in another series.^7^

We had no recurrent nerve palsy, hematoma or infection. The recurrent laryngeal nerve is systematically identified and preserved during the minimally invasive video-assisted thyroidectomy operation. Furthermore, the dissection of the upper thyroid pedicle is achieved with the external branch of the superior laryngeal nerve under direct view.^3^

The complication rate shown in the literature is similar to the rate for open surgery.^8^ In a series of 427 patients,^4^ there were complications caused by definitive recurrent nerve palsy in three patients (0.7%) and one case of definitive hypoparathyroidism (0.4%). In that series, wound infection was reported in three cases and there was no major bleeding that required surgical revision. Conversion to the open procedure was performed in five cases (1.2%).

It is appropriate to indicate the video-assisted approach for small thyroid papillary carcinomas because of the completeness achieved.^3^ Our two papillary carcinoma cases measured 6 and 8 mm. Previous results showed no statistical difference between minimally invasive video-assisted and conventional thyroidectomy, both in terms of 131-iodine uptake and circulating thyroglobulin levels.^9^ Total thyroidectomy is the standard protocol for well-differentiated carcinomas at our institution.

## CONCLUSIONS

The outcome from minimally invasive video-assisted thyroidectomy is good in terms of cosmetic results, analgesia and postoperative recovery. The scar is shorter than in the conventional procedure.
